# Postpartum Screening for Diabetes Among Women With a History of Gestational Diabetes Mellitus

**Published:** 2011-10-15

**Authors:** Alison Tovar, Lisa Chasan-Taber, Emma Eggleston, Emily Oken

**Affiliations:** John Hancock Research Center on Physical Activity, Nutrition, and Obesity Prevention, Friedman School of Nutrition Science and Policy, Tufts University; University of Massachusetts, Amherst, Massachusetts; Harvard Medical School and Harvard Pilgrim Health Care, Boston, Massachusetts. Dr Eggleston is also affiliated with the Brigham and Women's Hospital, Boston, Massachusetts; Harvard Medical School and Harvard Pilgrim Health Care, Boston, Massachusetts

## Abstract

**Introduction:**

To make recommendations for future clinical, public health, and research practices for women with abnormal glucose tolerance during pregnancy, we reviewed the latest evidence regarding rates of postpartum diabetes screening and types of screening tests.

**Methods:**

We searched PubMed for journal articles published from January 2008 through December 2010 that reported on postpartum screening and studies designed to prevent progression to type 2 diabetes among women with gestational diabetes mellitus (GDM). Two authors independently reviewed titles and abstracts from 265 articles.

**Results:**

From 34% to 73% of women with GDM completed postpartum glucose screening. Predictors of higher screening rates included older age, nulliparity, and higher income or education. Screening rates varied by race/ethnicity; Asian women were more likely to be screened than were other racial/ethnic minorities. Women who received prenatal care, who were treated with insulin during pregnancy, or who completed a 6-week postpartum visit were also more likely to receive screening. A moderate proportion of women screened had type 2 diabetes (1.2%-4.5%) or prediabetes (12.2%-36.0%).

**Conclusion:**

Rates of postpartum screening among women with a history of GDM are low; only half of women in most populations are screened. Our findings can inform future screening initiatives designed to overcome barriers to screening for both providers and patients. Well-designed lifestyle interventions specific to women with a history of abnormal glucose tolerance during pregnancy and also studies to determine the efficacy and safety of pharmacological interventions will be important to help prevent progression to diabetes among these high-risk women.

## Introduction

Type 2 diabetes mellitus, a global epidemic, affects approximately 21 million people in the United States (1). According to the 2005-2006 National Health and Nutrition Examination Survey (NHANES), the standardized total diabetes prevalence was 17.4% among both men and women, which reflects an absolute increase from the 1988-1994 NHANES of 3.7% among women compared with 2.1% among men (2). During this period, the number of women with undiagnosed type 2 diabetes increased, whereas the number of men with undiagnosed diabetes decreased. Given the damaging effect of prolonged undetected hyperglycemia, prevention and early diagnosis of type 2 diabetes is cost-saving and of public health importance (3).

Diagnosis of both gestational diabetes mellitus (GDM) and milder abnormal glucose tolerance in pregnancy helps health care professionals identify women who are at high risk for type 2 diabetes (4-7). GDM confers a 7-fold risk for future type 2 diabetes (8), and up to one-third of women with type 2 diabetes have been diagnosed with GDM (9). Although recommendations for postpartum screening of women with a history of GDM exist (3,10), many women are not screened. Furthermore, the most appropriate type and frequency of screening has recently been the subject of active discussion and investigation (11-13).

This article is part of the "Best Practices for Screening Reproductive Aged Women for Chronic Disease and Related Risk Factors" special collection in this issue of *Preventing Chronic Disease*. We review the latest evidence regarding postpartum screening rates and results among women with a history of GDM and also discuss findings from observational studies and randomized trials designed to prevent progression to type 2 diabetes among postpartum women with a history of GDM. In addition, we make recommendations for future clinical, public health, and research practice.

## Methods

### Data sources

To review postpartum screening rates and studies designed to prevent progression to type 2 diabetes among women with a history of GDM, we searched published journal articles in English from January 1, 2008, through December 31, 2010. We focused on the most recent literature to augment and update other recent reviews. We searched PubMed by using the search terms 1) "gestational diabetes" and "postpartum" or 2) or "gestational diabetes" and "follow-up." We also included additional articles that we identified by reviewing reference lists of studies and review articles.

### Study selection and data extraction

Our initial search returned 265 articles. Two authors (A.T. and E.O.) independently reviewed titles and abstracts and obtained all potential articles for additional review; they also conducted all data abstraction and reached consensus through discussion about any disagreement. Articles that were deemed irrelevant and excluded from the review were related to screening for GDM during pregnancy only, screening for metabolic syndrome or cardiovascular disease, or maternal or infant outcomes associated with GDM, type 1 diabetes, obesity during pregnancy, fertility and obstetric complications, interventions during pregnancy to improve GDM or improve pregnancy outcomes for mother or infant, or articles related to other diseases during pregnancy such as preeclampsia or polycystic ovary syndrome. Relevant articles reported on the following topics: 1) recommendations for types of screening tests, 2) rates and results of diabetes screening among women with prior GDM diagnosis at any time period postpartum, 3) results and comparisons of different types of screening tests, 4) predictors of and barriers to screening, and 5) interventions to improve rates of postpartum diabetes screening. We identified 11 published studies that evaluated rates and results of postpartum screening for diabetes among women with GDM (1-3) and 21 studies that discussed predictors of and barriers to screening or interventions to improve rates of postpartum diabetes screening (4,5). We discuss findings for the first postpartum year and the later postpartum period separately.

## Results

### Postpartum screening for type 2 diabetes among women with GDM

Both the American College of Obstetricians and Gynecologists (10) and the American Diabetes Association (3,14) recommend that women whose pregnancy was complicated by GDM be screened for persistent glucose abnormality at 6 to 12 weeks postpartum with either a fasting plasma glucose (FPG) test alone or with a fasting 75-g, 2-hour oral glucose tolerance test (OGTT). The OGTT is more sensitive, with reported sensitivities of 100% compared with 67% for FPG. The FPG test may be more acceptable to women because it requires less time, which could help overcome attrition in attendance for repeated follow-up testing in addition to being more easily tolerated (13,15). Criteria for diagnosing diabetes postpartum via a 75-g OGTT are similar to those for nonpregnant adult women (3), and thus results can identify overt diabetes mellitus, impaired fasting glucose (IFG), impaired glucose tolerance (IGT), or normal glycemia; however, the FPG test can identify IFG but not IGT. Both the American College of Obstetricians and Gynecologists and the American Diabetes Association recommend that women with a history of GDM with a normal postpartum screening be rescreened every 3 years, and women with IFG or IGT or both (prediabetes) should be rescreened annually. Recent consensus guidelines recommend follow-up testing with an OGTT at 1 year postpartum, FPG test yearly, and consideration of an OGTT at 3 years (16).

### Rates and results of postpartum screening for diabetes

We identified 11 studies published from 2008 through 2010 (17-27) that evaluated rates of postpartum screening for diabetes among women with GDM; of those, 8 were based on review of medical records and 3 were based on mailed questionnaires or interviews (Table). Together, these studies included 32,240 women with pregnancies complicated by GDM from 1999 through 2008. Postpartum diabetes screening rates varied but were generally poor: 34% to 73% (median, 48%) of women overall were screened.

Three of these studies included large, diverse populations of women receiving care at health maintenance organizations (HMOs) in California and Oregon affiliated with Kaiser Permanente. All 3 studies demonstrated substantial increases in screening during recent years. For example, among 14,448 women with GDM who received care at Kaiser Permanente Northern California, rates of postpartum screening increased from 20% in 1995 to 56% in 2006 (18). Even in the most recent years, however, fewer than 60% of women were screened. In 5 studies of smaller populations seen at referral centers in the United States (San Francisco, Texas), Canada, Poland, and Turkey, screening rates were similar (33.7%, 57.0%, 48.2%, 37.2%, and 47.4%, respectively) (17,22,23,26,27). Morrison et al surveyed Australian women whose mean (SD) duration since delivery was 21.2 (8.4) months. Respondents reported a higher overall screening rate of 73.2%, although only 27.3% reported having been screened according to Australian Diabetes in Pregnancy Society guidelines (a 75-g OGTT administered 6 to 8 weeks postdelivery) (24).

Although the recommended timing of screening is 6 to 12 weeks postpartum, all but 1 of the studies included longer follow-up (18). However, screening rates were not markedly improved when the time window was broader. For example in 2006, screening rates were 50.3% in a study within 3 months of postpartum follow-up (20), 48.2% to 51.0% in 2 studies within 6 months of postpartum follow-up (19,22), and 55.9% in a study within 12 months of postpartum follow-up (18). Among women seen at Kaiser Southern California who were screened within 6 months, 41% were screened from 7 days through 6 weeks postpartum, 46% from 6 through 12 weeks postpartum, and 12% from 12 weeks through 6 months postpartum (19).

The type of screening tests included and results varied across the studies. Ogonowski and Miazgowski and Swan et al included only OGTT (23,25). The other 9 studies included either FPG or OGTT (17-22,24,26,27) although, of these, 2 did not report percentages of women screened with each test (17,21). In 4 studies, 79% to 100% of women were screened with FPG (18-20,27), whereas in the remaining 3 studies, most women (56%-95%) were screened with OGTT (22,24,26) (Table). A number of women screened postpartum were found to have type 2 diabetes (1.2%-4.5%) or prediabetes (12.2%-36%) (Table). In Turkey, Kerimoglu et al reported that 50% (5 of 10) of the patients who received an OGTT and 7.4% (2 of 27) of the patients who received FPG were diagnosed with type 2 diabetes (27). Other studies have found that up to one-third of women with GDM will have abnormal postpartum screening (diabetes, IFG, or IGT) (28,29). According to Kwong et al, using only FPG in postpartum screening would cause up to 75% of women with IGT or type 2 diabetes to be missed (22). Similarly, Hunt et al found that of the women with postpartum OGTT with IGT, approximately one-third had outside range values on the 2-hour value alone (26). These findings are consistent with a substantial literature that a single FPG has a sensitivity of 16% to 89% when compared with an OGTT (13,15,30).

Three studies of postpartum screening rates used different methods and did not include test results. Almario et al identified women with GDM by using laboratory data from a university teaching hospital and found that only one-third of medical records included plans to perform postpartum screening (21). Morrison et al mailed questionnaires to Australian postpartum women with a history of GDM and found a higher rate of self-reported screening at any time (73.2%) compared with the 38%-45% elsewhere at the time of the study, although many fewer women (27.3%) received an OGTT within 6 to 8 weeks postpartum as per Australian recommendations (24). Another survey of 77 Australian women with a GDM pregnancy found that 61% self-reported postpartum diabetes screening (25).

### Factors associated with likelihood of postpartum diabetes screening

Rates of diabetes screening among postpartum women with a history of GDM differed by race and ethnicity, although findings varied (Table). For example, Stasenko et al found that Hispanic women had the lowest follow-up frequency (18% compared with 28% of white women and 29% of African American women) (17), whereas Lawrence et al found Hispanic women in another population were more likely to be screened (51% vs 48% of white women and 27% of African American women) (19). Asian women were most likely to be screened compared with women of other racial and ethnic backgrounds (odds ratio, 1.4; 95% confidence interval, 1.2-1.5) (18,19). Other patient characteristics associated with higher rates of postpartum screening included older age, nulliparity, and higher income or education. Women who received prenatal care, were treated with insulin during pregnancy, or completed a 6-week postpartum visit were also more likely to receive a postpartum diabetes screening (Table).

Of the 21 additional studies identified, 3 discussed barriers to postpartum screening (31-33). Barriers to postpartum screening included provider, patient, and system-level issues. In surveys of providers in Massachusetts, North Carolina, and Canada, providers commonly identified lack of patient attendance at a postpartum visit as the primary reason for not completing postpartum screening (31,32). However, in a chart review of data from 197 Massachusetts women with pregnancies complicated by GDM from 2000 through 2001, Smirnakis et al found that 94% of the women completed postpartum Papanicolaou screening, whereas 67% received any postpartum glucose testing and only 37% had a FPG or OGTT (34). Other barriers to postpartum screening identified by clinicians included 1) clinicians’ perception that screening guidelines were “inconsistent,” 2) patient cost or inconvenience (31), 3) lack of documentation of GDM on problem lists, and 4) poor communication between obstetricians and primary care providers (33). Patients identified time pressure as a primary barrier (32).

Postpartum reminders may help to improve diabetes screening rates. In a randomized trial of 223 women, mailed reminders resulted in improved screening rates within 12 months postpartum when sent to physicians only (52%), patients only (55%), or both (61%), compared with no reminders (14%) (35). However, only 48% of women seen at a Canadian diabetes referral clinic completed postpartum glycemic screening, even though women were given laboratory requisitions before delivery and received a telephone reminder if testing was not completed by 6 months postpartum (22). Ferrara et al reported that the proportion of women receiving an OGTT increased from 17% in 2005 to 72% in 2006, when Kaiser Permanente Northern California instituted a nurse-managed care program that included greater attention to postpartum screening guidelines (18).

### Factors associated with increased likelihood of abnormal postpartum screening test results

Women who have a family history of diabetes, are from a high-risk racial/ethnic group (eg, South Asian), have a higher prepregnancy or postpartum body mass index (BMI) (>25 kg/m^2^), have a higher glucose or greater number of abnormal glucose values on prenatal glycemic screening, who are diagnosed with GDM earlier in pregnancy, or who require insulin during pregnancy are more likely to have prediabetes or type 2 diabetes on postpartum screening (7,13,28,36-41). Fewer studies have examined potentially modifiable characteristics such as maternal diet (total energy intake, fat intake), physical activity, and breastfeeding (37), but these studies suggest that certain dietary patterns may play a role in increasing risk for developing type 2 diabetes.

### Diabetes screening after the early postpartum period

Risks for abnormal glycemia persist even after the early postpartum period. In a study by Retnakaran et al, among 70 women with GDM but normal OGTT at 3 months postpartum, 17% had prediabetes (IFG, IGT, or both) when reevaluated with OGTT at 12 months postpartum, compared with 3% of 73 women with normal glucose tolerance both during pregnancy and at 3 months postpartum (4). Even women with lesser degrees of abnormal glucose during pregnancy were at elevated risk for abnormal 12-month OGTT despite a normal 3-month OGTT (39). Risk factors for abnormal 12-month screening included higher 3-month postpartum glucose, BMI, low density lipoprotein cholesterol, triglycerides, and leptin, and lower high density lipoprotein cholesterol and adiponectin (4).

Risks for prediabetes, type 2 diabetes, and metabolic syndrome continue to increase over time, since pregnancy. Recent studies found that 16% to 30% of women with GDM develop type 2 diabetes by 5 to 10 years postpartum (42-44), and more cases accumulate even later. In a 2009 meta-analysis of 20 studies that included 675,455 women and 10,859 type 2 diabetes events, women with a GDM pregnancy had a nearly 7.5-fold increased risk of developing diabetes (8). Within 5 years of a pregnancy complicated by GDM, women had a relative risk of 4.7, which increased to 9.3 in those who were examined more than 5 years postpartum. In a 2010 population-based cohort study of more than 6,000 women from northern Finland followed since their pregnancies in 1986, the cumulative incidence of diabetes continued to increase even at 20 years postpartum. Rates were particularly high among women with a history of GDM who were overweight (26%), compared with controls who had no risk factors for GDM (0.7%) (45). Risk factors for late progression to type 2 diabetes are similar to risk factors for early progression and include low insulin sensitivity, insulin resistance and progressive insulin secretory defect, and gain in weight and body fat.

Although we identified several recent screening studies completed within the first postpartum year, we found no recent studies that evaluated rates of diabetes screening in clinical settings after this time period. We found only 1 published intervention to promote longer-term follow-up of women with a history of GDM; in South Australia beginning in 2002, women with GDM were invited to provide contact information to a GDM registry, which then sent women annual mailed reminders for glycemic screening and requests for diabetes screening updates beginning at 15 months postpartum (46). Findings suggest that these reminders helped promote screening. While 56% of women reported glycemic screening in the first postpartum year (before the intervention began), 75% reported having been screened during the second postpartum year. However, fewer than half of eligible women responded to the update requests, and thus reporting bias was likely.

## Discussion

Because the prevalence of type 2 diabetes is increasing among all groups in the United States and undiagnosed cases are particularly high among women, prevention and early diagnosis of type 2 diabetes have become public health priorities. A diagnosis of GDM identifies women who are at high risk for developing type 2 diabetes. We found that even recent studies published from 2008 through 2010 documented poor rates of postpartum screening for diabetes among women with pregnancies complicated by GDM.

Most of the studies we reviewed included follow-up periods longer than the recommended 6 to 12 weeks postpartum. Nevertheless, screening rates were low. This finding coincides with results from other studies, which suggest that some barriers to screening (eg, lack of patient attendance, inconsistency of screening guidelines, patient cost or inconvenience [32], and poor communication between obstetricians and primary care providers [33]) may persist beyond the recommended screening timeframe. Since risks for abnormal glycemia persist even after the early postpartum period (4,39), ongoing screening is required for women with a history of GDM every 1 to 3 years depending on the results of the postpartum screen and the likelihood of a future pregnancy. Although we found no recent studies that evaluated clinical screening for diabetes after the first postpartum year, rates of screening over the long term are probably even lower than during the early postpartum period. Further studies will be needed to evaluate the prevalence, predictors, and outcomes of long-term screening.

According to Lawrence et al, Kwong et al, and Hunt and Conway, many women with IGT or type 2 diabetes were likely missed when women were screened by using only an FPG test (19,22,26). This finding is consistent with earlier studies that found that the FPG test alone has poor sensitivity for diagnosis of prediabetes and type 2 diabetes compared with OGTT (30); however, its greater convenience may result in higher screening rates (47). Nevertheless, in 2007, Kim et al simulated postpartum screening for diabetes with FPG, OGTT, and hemoglobin A1c annually, every 2 years, and every 3 years over a period of 12 years, and found that OGTTs resulted in lower costs per case detected than FPG or hemoglobin A1c testing (48). More recently, the American Diabetes Association has allowed for the use of hemoglobin A1c as a screening test among the general population at risk for diabetes (3). Whether this test will be recommended for early postpartum screening remains unclear, but further research in this area is needed as the convenience and low participant burden of the hemoglobin A1c has the potential to increase screening rates.

Our review confirms that type 2 diabetes is common among postpartum women with a history of GDM and that screening is acceptable to many women. According to previous studies, screening is appropriate only if an effective intervention exists and if early identification and treatment result in improved outcomes (49). Findings from intervention studies, most notably the Diabetes Prevention Program (DPP), provide evidence that lifestyle changes or pharmacotherapy can prevent the onset of type 2 diabetes among women with a history of GDM who have abnormal test results (IFG or IGT) on postpartum screening (50-56). However, the lifestyle intervention in the DPP was intensive and expensive, limiting its generalizability. Additionally, women with a GDM history entered the DPP an average of 12 years postpartum. Given the demands of parenting an infant, it is likely that few women in the early postpartum period could engage in such an intensive program, yet early interventions are important. The first few postpartum years represent a particularly high-risk period for development of prediabetes or type 2 diabetes.

Randomized controlled trials of postpartum lifestyle interventions have not targeted women with GDM in particular, yet a recent systematic review found that 6 of 8 lifestyle interventions were effective at promoting postpartum weight loss (57). Recent studies (25,33,58) suggest that ample opportunity exists to improve weight and related lifestyle behaviors among postpartum women with a history of GDM. In addition to the traditional behavior change targets of diet and physical activity, observational studies suggest that breastfeeding, adequate sleep, and avoidance of television viewing may also help minimize postpartum weight retention and diabetes risk (59-62).

We identified no studies of postpartum screening for diabetes among women with GDM that also included information on prepregnancy or early pregnancy glycemic status. Whereas historically GDM has been defined as "any degree of glucose intolerance with onset or first recognition during pregnancy" (63), more recently it has been recognized that this definition includes a spectrum of glycemia entering pregnancy, including women with pregestational prediabetes or type 2 diabetes and also those with transient abnormal glucose levels that resolve after delivery. As more women become pregnant at older ages and with higher weights, a fraction of women diagnosed with GDM may actually have undiagnosed prediabetes or overt type 2 diabetes (64-66). Even women with abnormal glucose levels during pregnancy that do not meet criteria for GDM are at increased risk for postpartum prediabetes and type 2 diabetes (7,36). Recent consensus guidelines recommend first-trimester screening among women at high risk as well as thresholds that allow for diagnosis of overt diabetes at the time of early third trimester screening, which will identify women who merit especially close postpartum follow-up (67). As of 2011, these guidelines are endorsed by the American Diabetes Association (14). Future studies with information on women's glycemic status throughout the reproductive years will be invaluable in understanding the effect of abnormal glucose on outcomes during and after pregnancy.

There were several limitations to this systematic review. Because most of these studies were conducted within a large HMO system, where structural interventions for follow-up are often in place, or in diabetes referral clinic populations, postpartum screening rates are probably even lower among the general population. Also, 2 of the identified studies that reported higher screening rates were completed in Australia, a country with a different health care system from that of the United States. Newly recommended guidelines for GDM diagnosis will identify a much larger number of US women, perhaps 1 in 5 pregnant women, who will require treatment during pregnancy and screening postpartum (67). Future research is needed to assess the success of structural interventions in increasing postpartum screening rates in settings other than large HMOs, and also other intervention strategies such as those delivered directly to women via Internet, cell phone, or other technologies. Proven, cost-effective, and readily generalizable strategies to promote both early and ongoing postpartum diabetes screening among women with a history of GDM are greatly needed.

In conclusion, as rates of type 2 diabetes continue to increase, especially among women, more prevention opportunities are needed. Among women who experience glucose abnormalities during pregnancy, screening during the postpartum period offers a window of opportunity for early identification of diabetes and prediabetes. Although rates of screening have increased during the past several decades, these rates are still not optimal and need to increase, given the type 2 diabetes epidemic faced by both developed and developing nations. Increasing rates of screening may be challenging; different players (health care providers and public health workers) need to be involved. Future work should focus on reducing barriers to screening for both providers and patients. Lifestyle interventions specific to women with history of GDM are needed, as are studies to determine the efficacy and safety of pharmacological interventions among women of childbearing age.

## Figures and Tables

**Table.** Studies of Postpartum Screening for Diabetes Among Women With a History of Gestational Diabetes Mellitus (GDM), Published 2008-2010

**Reference**	**Study Design and Setting**	**Women Screened, %**	**Type of Screening, Results**	**Predictors of Screening**
Kerimoğlu et al, 2010 (27)	Telephone interview, n = 78 (of 335), identified from retrospective chart review Etlik, and Ankara, Turkey, 2005-2007	Overall, 47.4.	FPG (73%) or IGT (27%).Up to 12 wks postpartum. Results of OGTT: 50% T2DM, 20% IFG/IGT. Results of FPG: 7.4% T2DM, 40.7% IFG/IGT.	Insulin treatment during pregnancy and higher education (for OGTT screening only).
Swan 2010 et al, (25)	Mailed questionnaires, n = 84 (of 210), identified from regional referral hospitals medical record database in Victoria, Australia, 2001-2005	Overall, 61.0.	OGTT	Women living in small rural areas.
Stasenko et al, 2010 (17)	Retrospective chart review, n = 745, academic medical center, 2002-2008	Overall, 33.7. White, 27.7; African American, 28.6; Hispanic/Latina, 17.8; Asian, 43.2.	FPG or OGTT (% not reported), 0-6 mos postpartum; results: 2.0% T2DM, 28.3% IFG/IGT.	Older age, nulliparity, insulin requirement during pregnancy.
Lawrence et al, 2010 (19)	Retrospective chart review, n = 11,825, HMO, 1999-2006	Overall, 50.2. 1999: 34.4; 2006: 51.0. White, 47.7; Black, 27.2; Hispanic/Latina, 51.1; Asian, 59.0; Other/Unknown, 47.8.	FPG (79.1%) or OGTT (18.2%), both (2.7%). 7 d-6 mos postpartum. Results overall: 1.1% T2DM, 16.3% IFG/IGT. Results of OGTT: 2.3% T2DM, 27.6% IFG/IGT.	Older age, higher income, higher education, foreign-born, lower parity, vaginal delivery, nonmacrosomic infant, having a postpartum visit, having GDM coded diagnosis code, receiving no therapy or insulin (vs oral therapy alone).
Ferrara et al, 2009 (18)	Retrospective chart review, n = 14,448, HMO, 1995-2006	Overall, 38.2. 1995: 20.3; 2006: 55.9. White, 33.2; Asian, 45.5; Hispanic, 40.5; African American, 26.1; Other/Unknown, 34.1.	FPG or OGTT (1995, 5%; 2006, 71.5%). 6 wks-1 y postpartum. 2006 results: 2.7% T2DM, 31.3% IFG/IGT.	Older age, not being obese, lower parity, higher education, GDM diagnosis earlier in pregnancy, use of diabetes medications during pregnancy, more provider contacts after delivery.
Kwong et al, 2009 (22)	Retrospective chart review, n = 909, gestational diabetes clinic in Canada, 1999-2006	Overall, 48.2. Caucasian, 46.7; Non-Caucasian, 50.1.	FPG (4.8%) or OGTT (95.2%). 6 wks-6 mos postpartum. Results: 3.2% T2DM, 17.0% IFG/IGT.	Older age, lower parity, and insulin use during pregnancy.
Morrison et al, 2009 (24)	Mailed questionnaires, n = 1,372 (of 15,893), women registered with Australian National Diabetes Service Scheme, 2003-2005	Any screening, 73.2. Any screen 6-8 wks: 60.9. OGTT at 6-8 wks: 27.3.	FPG (32.6%), OGTT (56.4%). Any and within recommended 6-8 wks postpartum. Results not reported.	NA
Ogonowski and Miazgowski, 2009 (23)	Retrospective chart review, n = 855, diabetes clinic population, Poland, 2005-2007	Overall, 37.2.	OGTT 5-9 wks postpartum. Results: 1.3% T2DM, 12.2% IFT/IGT.	Older age, insulin requirement in pregnancy.
Dietz et al, 2008 (20)	Retrospective chart review, HMO population, n = 1,127 with confirmed GDM, 1999-2006	Overall, not reported. 1999: 9; 2006: 50.3.	FPG 100%, OGTT 0%. 0-3 mos postpartum. Results: 2.1% T2DM, 10.7% IFG.	NA
Almario et al, 2008 (21)	Retrospective review of laboratory records, university hospital, n = 90, 2004-2006	Screening tests ordered, 20.0.Screening test ordered or referred elsewhere for testing, 33.3.	FPG or OGTT (% not reported). 5-12 wks postpartum. Results not reported.	GDM diagnosis <24 wks gestation, GCT result >190 mg/dL, treatment of GDM with insulin or glyburide, family history of DM.
Hunt and Conway, 2008 (26)	Prospective cohort of predominantly Mexican or Mexican American women, n = 707, University Hospital of San Antonio, 2001-2003	Overall, 57.	FPG (28%) or OGTT (72%). 4-12 wks spostpartum. Results: 4.5% T2DM, 36% IFG/IGT.	Less likely to have had GDM, lower prepregnancy weight, lower point estimates for all glucose levels at diagnosis of GDM, nonuse of insulin or medications and more control over GDM.

Abbreviations: FPG, fasting plasma glucose; IGT, impaired glucose tolerance; OGTT, 2-hour, 75-g oral glucose tolerance test; T2DM, type 2 diabetes mellitus; IFG, impaired fasting glucose; HMO, health maintenance organization; NA, not applicable; DM, diabetes mellitus.
